# HS3ST2 expression induces the cell autonomous aggregation of tau

**DOI:** 10.1038/s41598-022-13486-6

**Published:** 2022-06-27

**Authors:** M. B. Huynh, N. Rebergue, H. Merrick, W. Gomez-Henao, E. Jospin, D. S. F. Biard, D. Papy-Garcia

**Affiliations:** 1 Glycobiology, Cell Growth and Tissue Repair Research Unit (Gly-CRRET), Univ Paris Est Creteil (UPEC), F-94010 Creteil, France; 2 Departamento de Bioquímica, Laboratorio Internacional Gly-CRRET-UNAM, Universidad Nacional Autónoma de México, Ciudad de México, México; 3 CEA, Institut de Biologie François Jacob (IBFJ), SEPIA, Université Paris-Saclay, Fontenay-aux-Roses, France

**Keywords:** Alzheimer's disease, Neurodegeneration, Alzheimer's disease, Biochemistry, Cell biology, Neuroscience, Diseases

## Abstract

Heparan sulfates have long been known to intracellularly accumulate in Alzheimer’s disease neurons, where they colocalize with neurofibrillary tangles made of abnormally phosphorylated and aggregated tau protein. However, the reasons and consequences of the heparan sulfates accumulation in the Alzheimer’s cells are not yet well understood. Previously, we showed that the neural heparan sulfate 3-*O*-sulfotransferase HS3ST2 is critical for the abnormal phosphorylation of tau in Alzheimer's disease-related tauopathy. Using cell models of tauopathy we showed that intracellular 3-*O*-sulfatated heparan sulfates interact with tau inducing its abnormal phosphorylation. However, it is unknown whether HS3ST2 expression induces the intracellular aggregation of tau in cells. Here, by using replicative pEBV plasmids, we engineered HEK293 cells to stably express HS3ST2 together with human tau carrying or not the P301S mutation. We show that HS3ST2 gain of function induces the cell autonomous aggregation of tau not only in cells expressing tau_P301S_, but also in cells expressing the wild type tau. Our engineered cells mimicked both the HS intracellular accumulation observed in neurons of Alzheimer’s disease and the tau aggregation characteristic of tauopathy development and evolution. These results give evidence that the neural HS3ST2 plays a critical role in the cell autonomous self-aggregation of tau.

## Introduction

Accumulation of the abnormally phosphorylated and aggregated microtubule associated protein tau (MAPT, or tau) is a main hallmark of Alzheimer's disease (AD) and other tauopathies^1–3^, including frontotemporal dementia with parkinsonism linked to chromosome 17 (FTDP17), progressive supranuclear palsy (PSP), corticobasal degeneration (CBD), Pick’s disease (PiD), and chronic traumatic encephalopathy. In the normal brain, the wild type tau promotes assembly and stability of microtubules^4^ and plays crucial roles in axoplasmic transport^5,6^, axonal outgrowth^7^, and synaptic plasticity^8^, centrally participating to the processes involved in learning and memory^9^. In the AD brain, the abnormally phosphorylated wild type tau (P-tau) becomes insoluble, aggregates, and accumulates to form neurofibrillary tangles (NFTs)^10^. A similar process occurs in tauopathies in which tau mutations induce the NFT formation^10,11^. Because NFTs accumulation correlates with neuronal degeneration^11^, haltering the aggregation of tau is currently considered among the main therapeutic strategies for AD and related tauopathies^12^. However, the endogenous players involved in the cell autonomous process leading to the primary self-aggregation of tau are not yet fully identified^13^. Interestingly, *in vitro*, tau aggregation can efficiently be promoted by anionic micelles and polyanions, including heparin, suggesting that an anionic micro-environment might play crucial roles in the genesis and maturation of tau aggregates^14–16^. Heparin is a commercially available animal sourced prototype of highly sulfated heparan sulfates (HS)^17^. A structural characteristic of heparin is that it carries a high content of 3-*O*-sulfation in addition to the classic *N*-, 2-*O*-, and 6-*O*-sulfations carried by common HS^18^. In the brain, 3-*O*-sulfation is assured by different HS 3-*O*-sulfotransferases (HS3STs), including the predominantly neural 3-*O*-sulfotransferase-2 (HS3ST2)^19–21^. However, whether cellular HS, and particularly 3-*O*-sulfated heparan sulfates (3S-HS), can participate to the processes leading to tau aggregation in cells remains elusive^22^. This is possibly because tau primarily aggregates inside cells whereas HS are generally considered to be located at the extracellular space^23,24^, restricting research commitment in this area. However, HS have been found to colocalize with tau helical filaments inside AD neurons, in where they accumulate before NFT formation^25–27^. By using cell models of tauopathy, we previously showed that 3S-HS accumulate at the intracellular level, interact with tau, and induce the tau abnormal phosphorylation^28^. However, it is unknown whether 3S-HS can promote the spontaneous self-aggregation of tau in cells.

Here, by simultaneously expressing HS3ST2 with human full length wild type tau (tau) or tau carrying the P301S mutation (tau_P301S_) responsible of FTDP17^11^, we demonstrate that 3S-HS produced by HS3ST2 induce the cell autonomous oligomerisation of tau and increase that of tau_P301S_. Immunocytochemistry (ICC) and immunoblotting of tau and P-tau after high salt sarkosyl extraction showed that, in the presence of HS3ST2, the wild type tau self-aggregates at extents near to those observed in the tau_P301S_, indicating that 3S-HS made by HS3ST2 can efficiently promote the aggregation of human tau in the absence of tau mutations. Finally, we confirmed that 3S-HS intracellularly accumulate and colocalize with tau and showed that the 3S-HS co-stain with tau oligomers in cells expressing HS3ST2 and tau, as in cells expressing tau_P301S_. The simultaneous and stable expression of HS3ST2 and tau proteins in cultured HEK293 cells allowed the demonstration that 3S-HS participate to the mechanisms triggering the spontaneous cell autonomous aggregation of tau.

## Materials and methods

### Plasmids

Plasmids carrying cDNA sequences allowing expression of the full length human tau and tau carrying the mutation P301S (tau_P301S_) in bacteria were kindly provided by Professor M. Goedert (University of Cambridge)^29^. Human full length HS3ST2 (NM_006043) was synthetized (Eurofins, France) and cloned in pcDNA^TM^3.1 vectors (Invitrogen) for protein expression in bacteria. For simultaneous expressions of HS3ST2 and tau, or tau_P301S_, the corresponding cDNA sequences were included in replicative pEBV plasmids containing single transcription cartridges for expression under hygromycin (HS3ST2) and puromycin (MAPT or MAPT_P301S_) selection^30,31^. Alternatively, a bicistronic vector carrying both the HS3ST2 and MAPT or MAPT_P301S_ sequences were constructed (Supplementary Method 1)^32^, but its use was restricted to few confirmatory studies because of risk of HS3ST2-tau fusion protein expression. Human cytomegalovirus (CMV) promoter sequence was included to drive transcription^30,31^. Construct sequences were confirmed by DNA sequencing (Eurofins Genomics, data not shown).

### Stable expression in HEK293 cells

Human embryonic kidney (HEK293) cells (provided by Dr. A. Delaunay, CEA Saclay, France) were cultured in Dulbecco’s Modified Eagle Medium (DMEM, Sigma) supplemented with 10% foetal calf serum (FCS), 100 U/mL penicillin, 100 µg/mL streptomycin, 2 mM glutamine, 10 mM HEPES (Sigma products), under 5% CO_2_. Approximately 100,000 cells were transfected with the agent JetPrime (Ozyme, following manufacturer’s instructions) to express HS3ST2 under hygromycin (100 µg/mL) selection. After HS3ST2 expression was confirmed, cells were additionally transfected with tau or tau_P301S_ and maintained under both hygromycin and puromycin selection (100 µg/mL and 0.4 µg/mL respectively). The empty pEBV vectors were used to follow effects of transfection and selection on cells. For Sarkosyl experiments, 10^6^ cells were transfected. From 24 h after second transfection, cells were permanently maintained under hygromycin/puromycin selection until stabilisation (40 days in culture).

### RNA extraction and RTqPCR

RNA extraction was performed with RNA-Bee (Bioconnect) following manufacturer’s recommendations. RNA concentration was measured with a Nanodrop 2000 (Thermoscientific). After a DNAse treatment (Ambion), according to manufacturer’s procedures, 1 µg RNA was used to synthesize complementary DNA (cDNA) with the Affinity Script Multiple Temperature cDNA kit (Agilent). Samples were incubated at 25 °C for 10 min, at 50 °C for 60 min, and at 70 °C for 15 min. Gene expression was analysed in template cDNA by quantitative real time polymerase chain reaction (qPCR) using the Brilliant III Ultra-fast SYBR^*^ kit (Agilent Technologies) in an Agilent AriaMx thermocycler. Analysis was performed with the Agilent AriaMx 1.0 software. Primer sequences for HS3ST2 were “GGA ACC CCA CTT CTT TGA CA” and “GTC GAG GAG CCT CTT GAG TG”^33^. Primer sequences for MAPT were “AAG GTC AGC TTG TGG GTT TC” and “TGG CTC ATT AGG CAA CAT CC” (Eurofins). Primer sequences for B2M were “GCT CCG TGG CCT TAG CTG T” and “ACG TGA GTA AAC CTG AAT CTT TGG A”. Primer sequences for RPL37A were “ATT GAA ATC AGC CAG CAC GC” and “AGG AAC CAC AGT GCC AGA TCC”. Other primers sequences used in this study (other HS3STs) are detailed in Supplementary Table S1. Standard curves were used for calculation of relative RNA quantity B2M and/or RPL37A were was used as housekeeping genes for normalization^34^.

### Immunoblotting of RIPA-extracted proteins

Cells were harvested in 1 mL of RIPA buffer (50 mM tris, pH 8.0, 150 mM NaCl, 0.1% Triton X-100, 0.5% sodium deoxycholate, 0.1% SDS; Thermo Fisher Scientific) supplemented with 1% protease inhibitor mixture (Sigma) and 1% phosphatase inhibitor cocktail I & II (Sigma). Cell lysates were centrifugated at 13,000 rpm for 10 min and protein contents in supernatants were determined using BCA Protein Assay kit (Thermo Fisher Scientific). Supernatants containing 1–10 µg of protein (as indicated) were suspended in Laemmli buffer (0.125 M Tris HCl, pH 6.8, 4% SDS, 20% glycerol, 0.004% bromophenol blue; Biorad) supplemented with 10% β-mercaptoethanol and heated for 5 min at 90 °C. Proteins were separated in 8% acrylamide gel and transferred to a PVDF membrane with a Transblot Turbo device (BioRad). Immunoblotting was performed with anti-HS3ST2 (Thermo Fischer Scientific, 1:500), and anti-total tau K9JA (Dako, 1:10,000). Anti-GAPDH (Thermo Fisher Scientific, 1:10,000) or anti-α-tubulin (α-tub, Sigma, 1:10,000) were used as loading control. Blots were incubated for 1 h at room temperature (rt) with the corresponding secondary antibodies diluted in PBS containing 5% milk. Revelation was performed with the Immobilon Western Chemiluminescent HRP Substrate Luminata Forte (Millipore) following manufacturer’s instructions.

### High salt Sarkosyl tau oligomers extraction and immunoblotting

To extract tau oligomers^35,36^, cells were harvested in 1 mL of salt rich Sarkosyl buffer (50 mM HEPES pH 7.0, 250 mM sucrose, 1 mM EDTA, 0.5 M NaCl, 1% sarkosyl) supplemented with 1% protease inhibitor mixture (Sigma) and 1% phosphatase inhibitor cocktail I & II (Sigma). Cell lysates were centrifugated at 180,000 g for 30 min and protein content in supernatants were determined using BCA Protein Assay kit (Thermo Fisher Scientific). Cell lysate supernatant containing 1–10 µg of proteins (as indicated) were treated or not (as indicated) in Laemmli buffer (Biorad) supplemented with β-mercaptoethanol. Samples treated with Laemmli buffer were heated for 2–5 min at 90 °C to denature tau oligomers. Proteins were separated by electrophoresis as above. Antibodies used for immunostaining were anti-total tau K9JA (Dako, 1:10,000), T22 (Millipore, 1:500), and PHF1 (from Peter Davis, 1:500), as indicated. Anti-GAPDH (Thermo Fisher Scientific, 1:10,000) or anti-α-tub (α-tub, Sigma, 1:10,000) were used as loading controls, as indicated. Blots were incubated with secondary antibodies and revealed as described above.

### Immunocytochemistry

For immunocytochemistry (ICC) experiments, approximately 50,000 cells were cultured on glass cover slips. After transfection and culture as indicated, cells were fixed in 100% methanol at −20 °C for 3 min and incubated for 1 h with the corresponding primary antibody at rt, washed, and then incubated with the appropriate secondary antibody for 1 h at rt. Immunostaining was performed with anti-HS3ST2 (Thermo-Fischer Scientific, 1:500), anti-total tau (K9JA Dako, 1:500), or anti-oligomeric tau (T22 Millipore; 1:700). Fluo488 donkey anti-rabbit (Invitrogen; 1:200) and Fluo488 donkey anti-goat (Invitrogen; 1:200) were used as secondary antibodies. The phage display HS4C3 antibody (1:250; 4 µg/mL), kindly provided by ArrestAD partner Pr. Toin H. van Kuppevelt (Radboud University Medical Center, the Netherlands) was used to detect 3S-HS^37^. To reveal HS4C3 staining, an anti-VSV antibody made in mouse (Sigma; 1:200) was used, followed by a Cy3 anti-mouse made in sheep (Sigma; 1:200). HS identity was confirmed by treating cells with a mix of heparinase I, II and III (Iduron) respectively used at 2, 0.2, and 0.2 U/mL. Stack images were obtained with the software CellSens from a spinning disk inverted confocal microscope (IX81 DSU Olympus, 60 × N.A.1.35) coupled to an Orca Hamamatsu RCCD camera. 20 × images were obtained with the same microscope in non-confocal configuration. Nuclei was labelled with 1 µg/mL DAPI (Sigma). Images were processed with the ImageJ software (W. Rasband, National Institute of Health).

### Statistical analysis

Statistical analyses were performed using GraphPad Prism software version 5.01. Bars represent standard error (SD) or standard error of the mean (SEM) for two or three different experiments each performed in duplicate or triplicate, as indicated. Significance was analysed either by t-test, or one-way ANOVA followed by Tukey’s test, as indicated (***P < 0.001; **P < 0.01; *P < 0.05; *ns* = not significant).

## Results

### HS3ST2 expressing cells produce 3S-HS

**Figure 1:**
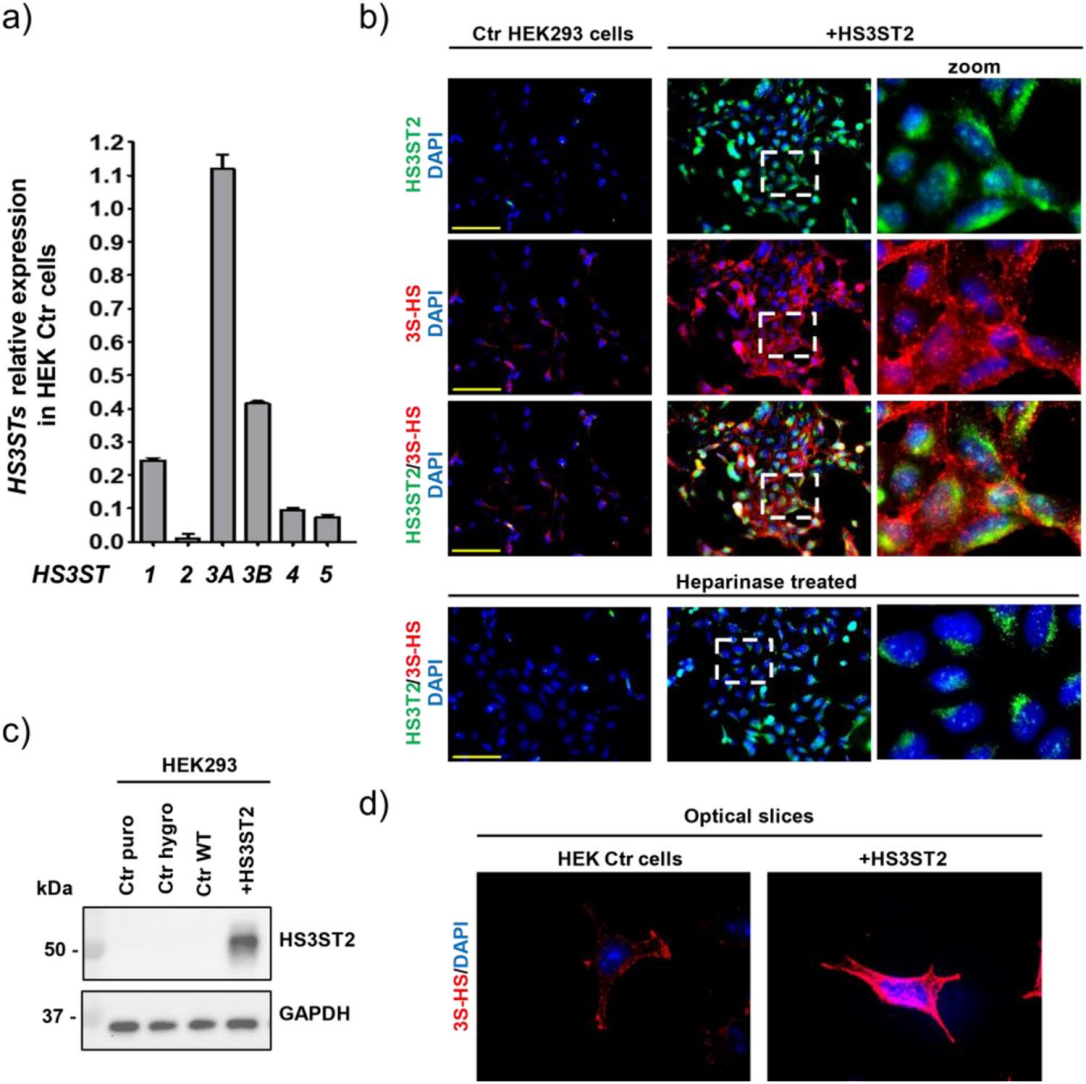
HEK293 expressing HS3ST2 cells produce 3S-HS. (**a**) Expression of different HS3STs in wild type (WT) HEK293 cells (HEK Ctr cells). Relative gene expression was normalized with B2M and RPL37A. Mean values ± SD (*n* = 3) are represented. (**b**) HS3ST2 and 3-*O*-sulfated HS (3S-HS) immunostaing in HEK Ctr cells and in HS3ST2 expressing cells (HEK+HS3ST2). The newly expressed HS3ST2 was enzymatically active, as shown by 3S-HS immunolabelling with the HS4C3 antibody loss of signal after heparitinase treatment (lower panel) in (**b**). Cells were counterstained with DAPI to visualize nuclei (blue). Images were acquired by a IX81 Olympus microscope (magnification 20x), scale bar is 100 µm. (**c**) HS3ST2 protein immunoblotting using GAPDH as loading control (RIPA cell lysates). (**d**) 3S-HS immunocytochemistry using the HS4C3 antibody shows staining predominantly at the cell membrane. Confocal slice images were acquired by a confocal microscope IX81 Olympus (magnification 60×, immersion oil).

Previously, we showed in cell models of tauopathy that HS3ST2 critically participates to the abnormal phosphorylation of tau^28^. However, it is unknown whether HS3ST2 expression can lead to the formation of tau aggregation in cells. To target this question, we chose to simultaneously express HS3ST2 and tau or tau_P301S_ in cells lacking these proteins or expressing them in very low extent. We chose to work with HEK293 cells (further referred as HEK Ctr cells) as the two proteins were reported to be absent, or lowly expressed in these cells (http://www.proteinatlas.org)^38^. By using RT-qPCR, we first confirmed low HS3ST2 and tau expressions levels in the HEK Ctr cells (Fig. 1a). HS3ST2 transcripts were the lowest compared to other HS3STs expressed in the HEK Ctr cells, suggesting that 3S-HS can be produced at some extent by other HS3STs in these cells. Accordingly, ICC and immunoblotting showed no detectable HS3ST2 protein in the HEK Ctr cells (Fig. 1b, c; full blots in Supplementary Fig. S1) whereas ICC with the anti 3S-HS antibody (HS4C3)^37^ showed some 3S-HS staining. As expected, confocal microscopy images confirmed that 3S-HS in the HEK Ctr cells are located at the cell membrane (Fig. 1d). This is in agreement with the well-established membrane location of HS under physiological conditions^23,24^. To validate that HS4C3 can stain 3S-HS produced by HS3ST2, we transfected HEK293 cells with the replicative pEBV plasmid carrying the sequence coding for the full length human HS3ST2 (cells are further referred as HEK+HS3ST2 cells). As expected, HS3ST2 immunostaining and immunoblotting, as well as 3S-HS staining, were stronger in the HEK+HS3ST2 cells compared to the HEK Ctr cells (Fig. 1b, upper and middle panels and Fig. 1c). Again, 3S-HS were observed in the cell membrane of HEK+HS3ST2 cells, although some signal was detected in the intracellular compartment (Fig. 1d). The strong 3S-HS staining in the HEK+HS3ST2 cells indicated that the newly expressed enzyme was active, as confirmed by loss of the HS signal after heparinase treatment of both Ctr and transfected cells (Fig. 1b, lower panel). Together, these results show that enzymatically active HS3ST2 can efficiently be expressed in HEK293 cells and that the produced 3S-HS are predominantly located on the cell membrane.

### HS3ST2 and tau do not induce their reciprocal expression

**Figure 2:**
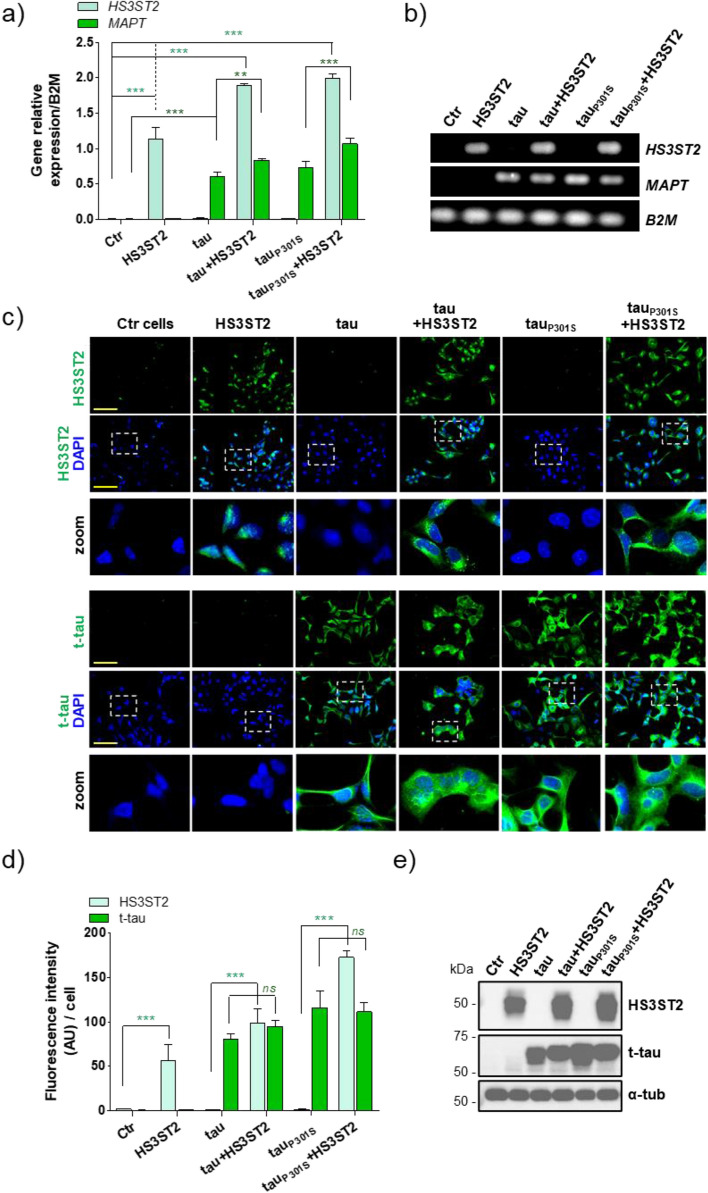
HS3ST2 and tau do not induce their reciprocal expression. HEK293 cells were transfected to independently or simultaneously express HS3ST2, tau, and/or tau_P301S_. (**a**) RTqPCR quantification of HS3ST2 and MAPT (tau) transcripts in the transfected HEK293 cells (Ctr). Relative gene expression was normalized with B2M. Mean values ± SD (*n* = 3) are represented. (**b**) Final point PCR quantification of HS3ST2 and MAPT (tau) transcripts in the transfected HEK293 cells (Ctr). B2M was used as loading control. (**c**) Immunostaining of HS3ST2 (anti-HS3ST2) and total tau (t-tau) (K9JA antibody) in HEK293 cells (Ctr) and transfected cells. Cells were counterstained with DAPI to visualize nuclei (blue). Images were acquired by a microscope IX81 Olympus, magnification 20 ×, scale bar 100 µm. (**d**) Relative fluorescence intensity of HS3ST2 and t-tau in engineered cells. (**e**) Immunoblotting of RIPA cell lysates with anti-HS3ST2 and K9JA (anti-t-tau) antibodies. Anti-α-tubulin (α-tub) antibody was used as loading control. Mean values ± SD (*n* = 3) are represented. One-way analysis of variance (ANOVA) was performed, followed by Tukey’s test, as indicated (***P < 0.001; **P < 0.01; *ns* = not significant).

To investigate whether HS3ST2 expression induces tau and tau_P301S_ expressions, and if tau or tau_P301S_ expression induces HS3ST2 expression, cells expressing the full-length human tau carrying or not the P301S mutation (further referred as HEK+tau or HEK+tau_P301S_ cells) and cells simultaneously expressing both HS3ST2 and tau carrying or not the tau mutation (further referred as HEK+tau+HS3ST2 or HEK+tau_P301S_+HS3ST2) were generated. RT-qPCR analysis showed that HS3ST2 and tau transcripts were only increased in cells transfected with the corresponding plasmids (Fig. 2a, b). HS3ST2 expression was only increased in the HS3ST2 transfected cells (HEK+HS3ST2, HEK+tau+HS3ST2, and HEK+tau_P301S_+HS3ST2), but not in the tau only expressing cells (HEK+tau or HEK+tau_P301S_). Similarly, tau expression was increased only in the tau transfected cells (HEK+tau, HEK+tau_P301S_, HEK+tau+HS3ST2, and HEK+tau_P301S_+HS3ST2), but not in the HS3ST2 only transfected cells (HEK+HS3ST2) (Fig. 2a, b). After assessing transcript levels, proteins were analysed by ICC and immunoblotting (WB) using anti-HS3ST2 and anti-total tau (K9JA) antibodies. As expected, both proteins were only detected in the corresponding transfected cells (Fig. 2c–e; complete blots are shown in Supplementary Fig. S2), indicating successful protein expression. Together, these results show that, by using pEBV plasmids, cells independently or simultaneously expressing HS3ST2 and/or tau can be generated without inducing their reciprocal expressions.

### HS3ST2 promotes tau and tau_P301S_ oligomerisation

**Figure 3:**
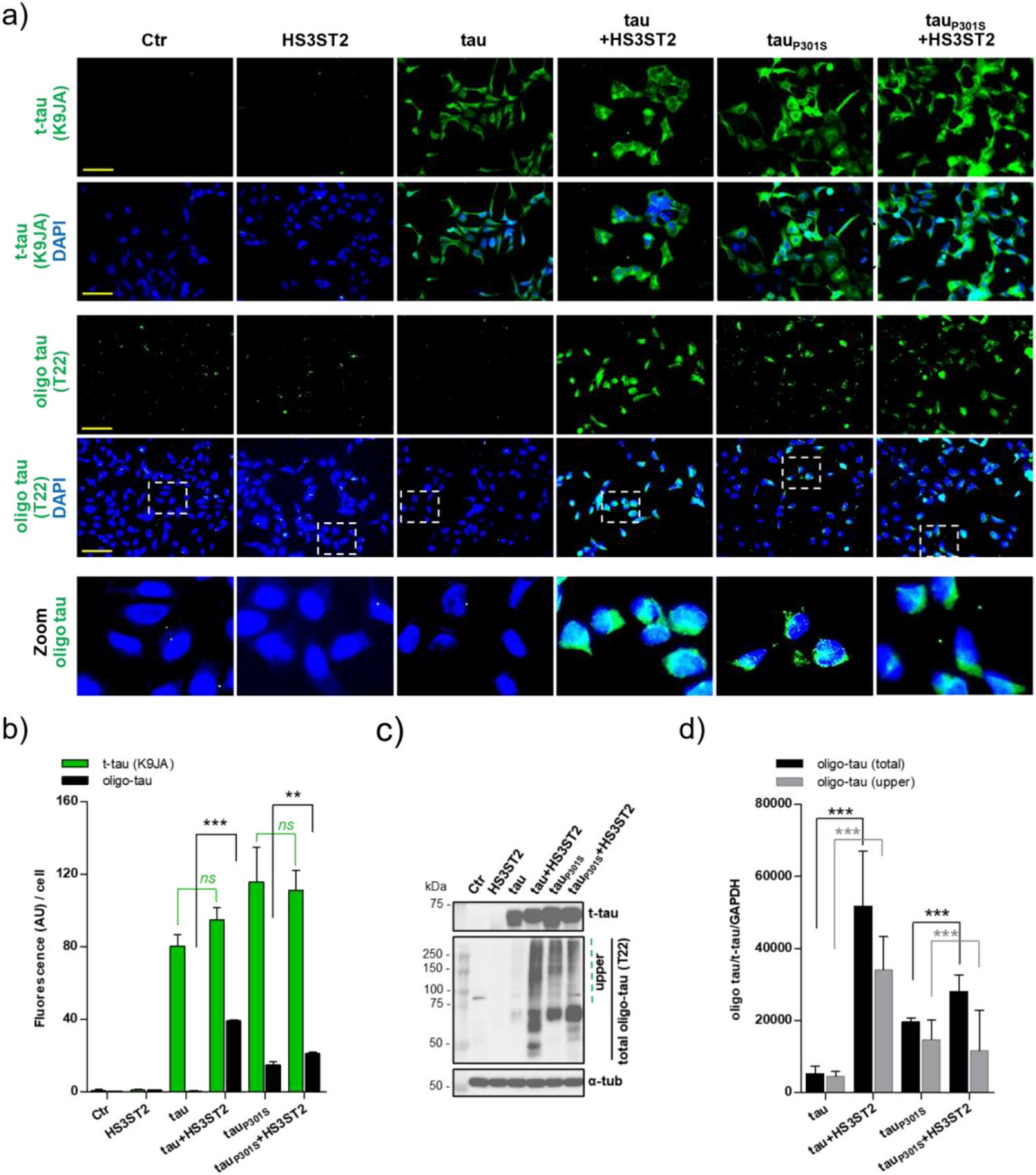
HS3ST2 promotes tau oligomerisation in cells expressing tau and tau_P301S_. (**a**) Total tau (t-tau) and oligomeric tau (oligo-tau) were respectively stained with the K9JA and T22 antibodies. Cells were counterstained with DAPI to visualize nuclei (blue). Images were acquired with microscope IX81 Olympus, magnification 20x, scale bar 100 µm. (**b**) Fluorescence intensity was quantified in cells by using ImageJ (2 fields, or 3 when cell density was low), t-tau signal (K9JA, green histograms) and oligo-tau signal (T22, black histograms) were compared between cells expressing or not HS3ST2. Mean values ± SD (*n* = 3) are represented. (**c**) T-tau (K9JA) immunoblotting (upper blot) was performed in RIPA cell lysates and proteins were detected in all the MAPT transfected cells. Oligo-tau (T22) immunoblotting (medium blot) was performed on cell lysates extracted with high salt (500 mM) Sarkosyl buffer. Stable tau aggregates are highlighted in the upper part of the blot (green dotted line). An anti-α-tubulin (α-tub) antibody was used as loading control (RIPA). (**d**) Oligo-tau and t-tau signals quantification were performed with ImageJ in the different cells (black histograms: oligo-tau/t-tau/GAPDH, grey histograms: upper oligo-tau/t-tau/GAPDH) and the average signal from two experiments was compared. Mean values ± SD (*n* = 3) are represented. Image or immunoblot analyses were calculated by one-way ANOVA with Tukey’s test, as indicated (***P < 0.001; **P < 0.01; *ns* = not significant). Experiments were reproduced twice with similar results.

After production of cells independently or simultaneously expressing HS3ST2 and/or tau or tau_P301S_, we investigated whether HS3ST2 expression can lead to the formation of oligomeric tau in these cells. With this aim, we used the T22 antibody, which specifically stains the oligomeric tau (oligo-tau) characteristic of early stages of tau aggregation, although staining decreases when the oligomeric tau reaches higher aggregation stages^39^. In our system, the HS3ST2 capacity to trigger the cell autonomous oligomerisation of non-mutated tau was suggested by T22 staining of tau oligomers, which were only detected when HS3ST2 was expressed with tau (Fig. 3a–d). The HS3ST2 capacity to trigger tau oligomerisation was confirmed with the mutated tau_P301S_ expressing cells, as an increased T22 staining was observed in HEK+tau_P301S_+HS3ST2 cells compared to the HEK+tau_P301S_ cells (Fig. 3a, b). As expected, HEK Ctr cells and HEK+HS3ST2 showed non-significant T22 staining compared to HEK Ctr cells. However, a decreased T22 signal level was observed in HEK+tau_P301S_+HS3ST2 cells compared to HEK+tau+HS3ST2 cells (Fig. 3b). This is possibly due to a decreased T22 immunoreactivity characteristic of advanced aggregation stages^39^. Accordingly, an increased propensity of HEK+tau_P301S_+HS3ST2 cells to trigger tau oligomerisation compared to HEK+tau+HS3ST2 cells was confirmed in bicistronic vectors transfected cells (Supplementary Method 1), which can be analysed at earlier times after transfection (Supplementary Fig. S3). However, the bicistronic vectors were not used in the continuation of the study because of the detection of fused HS3ST2-tau proteins (results not shown). These results suggest that HS3ST2 triggers the formation of oligomeric tau and boosts the oligomerisation of tau_P301S_ in cells.

Thus, tau oligomers were isolated using Sarkosyl salt-rich buffer extraction and analysed by immunoblotting^35,36^. The total tau signal detected by the K9JA antibody in RIPA protein extracts (Fig. 3c upper blot; full blots in Supplementary Fig. S4), was compared to oligo-tau signal detected by the T22 antibody in hight salt Sarkosyl extracts (Fig. 3c; middle blot, full blots in Supplementary Fig. S4). Oligo-tau levels were significantly higher in cells expressing tau and HS3ST2, compared to cells expressing tau but not the enzyme (Fig. 3d), regardless of similar expression of total tau (Fig. 3c, d). Similarly, higher oligo-tau levels were observed in cells expressing tau_P301S_ and HS3ST2 comparing to tau_P301S_ only expressing cells (Fig. 3c, d). As in ICC observations, a decreased T22 staining was observed when HEK+tau_P301S_+HS3ST2 cells were compared to HEK+tau+HS3ST2 cells (Fig. 3b–d), suggesting no immunoreactivity of T22 at advanced oligomerisation. These results opened to the question whether the observed decrease of tau oligomers was accompanied by an increase of tau hyperphosphorylation.

### HS3ST2 promotes tau and tau_P301S_ oligomerisation and hyperphosphorylation in cells

**Figure 4:**
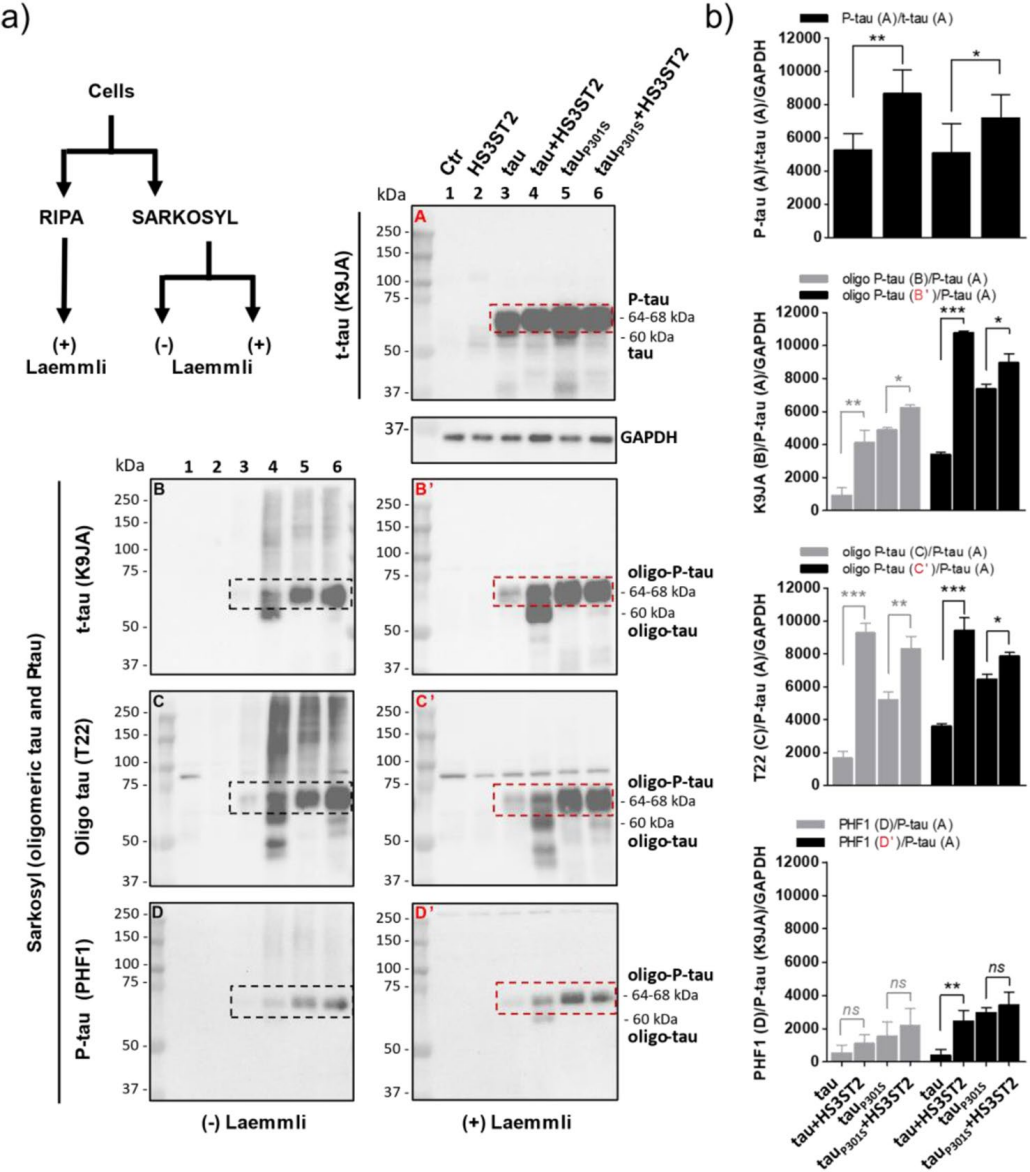
HS3ST2 promotes tau aggregation in tau expressing cells. (**a**) High-salt Sarkosyl and RIPA buffer protein extracts were analysed in cell lysates from control and transfected cells. Immunoblotting of RIPA (blot A) and high salt Sarkosyl buffer (blots B-D and B’-D’) extracted proteins were performed before and after protein denaturation by heating in Laemmli buffer (B-D and B’-D’, respectively). Total tau (K9JA, blot A), oligomeric total tau (blots B-C and B’-C’), and oligomeric tau hyperphosphorylated at serine 396/404 (PHF1 antibody, blots D and D’) were analysed before and after denaturation. (**b**) Total tau, oligomeric-tau (oligo-tau), and P-tau signal intensities were quantified by ImageJ. Mean values ± SD are represented. Immunoblot analyses were calculated by one-way ANOVA with Tukey’s test, as indicated (***P < 0.001; **P < 0.01; *P < 0.05; *ns* = not significant). Experiments were reproduced twice with similar results.

To investigate if in our transfected cells total tau and oligomeric tau and tau_P301S_ were hyperphosphorylated, we focused our attention in the immunoblot bands showing increased molecular weight before or after complete denaturation of Sarkosyl or RIPA extracted proteins^40^ (Fig. 4a). Sarkosyl is an ionic detergent which under high salt concentration (NaCl 500 mM) allows recovery of tau oligomers that can be separated from soluble tau and tau fibers, if present^35,36^. These tau oligomers can be detectable by the T22 antibody^39^. On the other side, extraction with RIPA allows soluble protein recovery, including soluble tau (but not fibrous tau aggregates)^40,41^, which together represent the total tau detectable with K9JA antibody. Accordingly, K9JA immunoblotting of RIPA cell lysates allowed clear detection of soluble t-tau in both tau and tau_P301S_ expressing cells (Fig. 4a, blot A; full blots in Supplementary Fig. S5) whereas in the high salt Sarkosyl extracted proteins, the K9JA tau signal intensity was dramatically reduced only in the HEK+tau cells (Fig. 4a, blots B-C and B’-C’; full blots in Supplementary Figs. S5 and S6). This confirms observation on Fig. 3a–c showing that the non-mutated tau can efficiently form oligomers in cells expressing tau and HS3ST2 but not in cells expressing the non-mutated tau only. Interestingly, tau was detected predominantly as two main bands, one around 60 kDa and a second at 64–68 kDa. Indeed, tau purified from the human brain migrates as ~ 50–60 kDa bands on SDS-gel due to the presence of six isoforms^40,42–45^. Here, only full-length tau (60 kDa) is expressed by the transfected cells. On the other side, PHF-tau isolated from AD brain displays characteristic 60–64 and 68 kDa bands on SDS gels. The 64–68 kDa bands have been shown to be hyperphosphorylated and are considered as a pathological marker of AD^40,42–45^. Here, the 64–68 kDa (P-tau) band was the only band detected in RIPA extracts from cells expressing tau or tau_P301S_ together with HS3ST2, whereas the 60 kDa band was also present in cells not expressing HS3ST2 (Fig. 4a, blot A). This suggests that HS3ST2 expression enhances the hyperphosphorylation of both tau and tau_P301S_ (Fig. 4a, blot A; full blots are shown in Supplementary Fig. S5). Accordingly, quantification of the hyperphosphorylated tau 64–68 band (Fig. 4b, graph A: P-tau/t-tau/GAPDH) confirms higher P-tau levels in the HS3ST2 expressing cells. Immunoblotting of oligomeric tau extracted by high salt Sarkosyl buffer showed that the 64–68 kDa P-tau is oligomerized in HEK+tau+HS3ST2, HEK+tau_P301S_ and HEK+tau_P301S_+HS3ST2 cells, but not, or less, in the tau only expressing HEK+tau cells (Fig. 4a, blot A *vs* blots B-D and B’-D’; full blots in Supplementary Figs. S5–S6), Again, this suggests that P-tau was oligomerized in HEK+tau+HS3ST2, HEK+tau_P301S_, and HEK+tau_P301S_+HS3ST2 expressing cells, but not in tau only expressing cells, in agreement with the strong T22 signal observed in Sarkosyl extracts from these cells (Fig. 4a, blots C and C’) and with T22 staining in Fig. 3. On the other side, the 60 kDa tau band was not extracted by high salt Sarkosyl either from HEK+tau+HS3ST2 cells nor from HEK+tau_P301S_ cells (Fig. 4a, blot A *vs* blots B-C and B’-C’), suggesting that tau was not oligomerized in these cells. However, the 60 kDa tau band appeared in tau-HS3ST2 expressing cells after Sarkosyl extraction, possibly because the protein was aggregated in the HS3ST2 expressing cells and thus not recovered in the RIPA buffer (Fig. 4a, blot A *vs* blots C and -C’). This is also suggested by the 60 kDa increased band density after oligomers denaturation by heating in Laemmli (Fig. 4a, blot A *vs* blots B-C and B’-C’). In line with these observations, tau (K9JA) band quantification shows increased P-tau oligomers in the two HS3ST2 expressing cells before and after cell lysate denaturation by Laemmli (Fig. 4b, graph B). Immunoblotting with the more specific anti-oligomeric tau T22 antibody showed similar oligomeric tau extent in the two cell lines expressing HS3ST2 (both before and after Laemmli denaturation; Fig. 4b, graph C). Thus, HS3ST2 can similarly induce tau oligomerisation of tau and tau_P301S,_ although the tau oligomers are less hyperphosphorylated in the HEK+tau+HS3ST2 cells compared to the HEK+tau_P301S_+HS3ST2 ones. Accordingly, analysis of the extracted oligomers with the anti-P-tau PHF1 antibody^46^, showed a less intense PHF1 signal at 64–68 kDa in tau only expressing cells compared to tau_P301S_ expressing cells. In both cases the PHF1 signal increased when HS3ST2 was expressed (Fig. 4a, blot D-D’ and Fig. 4b PHF1), confirming that HS3ST2 promoted tau hyperphosphorylation. Together, these results show that HS3ST2 expression triggers a cell autonomous hyperphosphorylation and oligomerisation of both non-mutated tau and tau carrying the pathogenic mutation P301S.

### 3S-HS intracellularly accumulate in cells producing tau and tau_P301S_ oligomers

**Figure 5:**
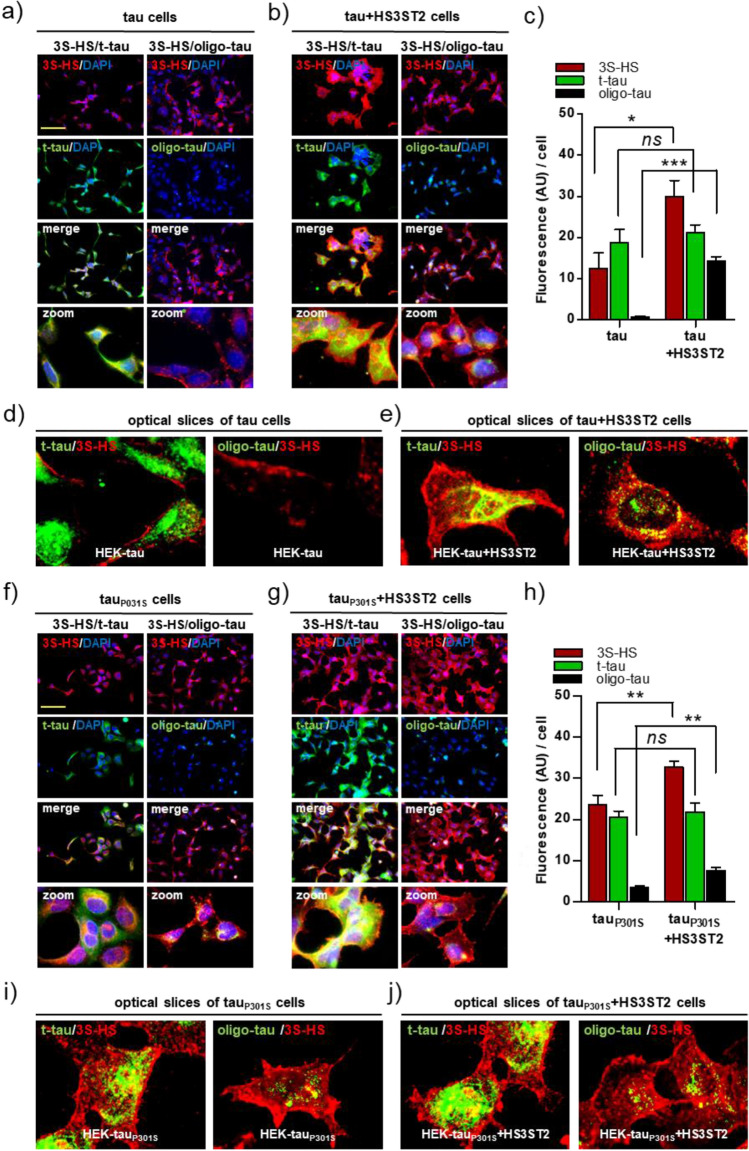
Intracellular 3S-HS and tau oligomers in cells expressing HS3ST2 and tau or tau_P301S_. Cells were immunoassayed with antibodies against 3S-HS (HS4C3, red), total tau (t-tau; K9JA antibody; green), and oligo-tau (T22 antibody; green). Images were recorded with a microscope IX81 Olympus with confocal option. Magnification was 20× or 60× for confocal slices (immersion oil). (**a,b**) Representative image of 3S-HS, tau, and oligo-tau staining in HEK cells expressing tau together or not with HS3ST2. (**c**) Fluorescence intensity quantification of 3S-HS, t-tau, and oligo tau signals in HEK+tau *vs* HEK+tau+HS3ST2 cells. Mean values ± SD (*n* = 3) are represented. (**d**) Confocal slices showing non-intracellular 3S-HS and no tau oligomers in HEK+tau cells. (**e**) Confocal slices showing intracellular 3S-HS and tau oligomers accumulation in HEK+tau cells. (**f,g**) Representative images of 3S-HS, tau, and oligo-tau staining in HEK cells expressing tau_P301S_ together or not with HS3ST2. (**h**) Fluorescence intensity quantification of 3S-HS, t-tau, and oligo tau_P301S_ signals in HEK+tau_P301S_ *vs* HEK+tau_P301S_+HS3ST2 cells. Mean values ± SD (*n* = 3) are represented. (**i**) Confocal slices showing intracellular 3S-HS and tau oligomers in HEK+tau_P301S_ cells. (**j**) Confocal slices showing intracellular 3S-HS and tau oligomers in HEK+tau_P301S_ and HEK+tau_P301S_+HS3ST2 cells. Image analyses were calculated by the unpaired and two-tailed Student’s t test, as indicated (***P < 0.001; **P < 0.01; *P < 0.05; *ns* = not significant).

Here, we showed that the cell autonomous hyperphosphorylation and oligomerisation of tau and of tau_P301S_ is induced in cells expressing HS3ST2. Previously, we showed in SH-SY5Y cell models of tauopathy that 3S-HS accumulate and colocalize with tau at the intracellular level^28^. To investigate whether 3S-HS intracellularly accumulate and colocalize with tau in our engineered cells producing hyperphosphorylated tau oligomers, we stained cells with total-tau (K9JA), oligo-tau (T22), and 3S-HS (HS4C3) antibodies and analysed cells by ICC with confocal microscopy. As expected, tau was intracellularly located in all tau expressing cells, whereas 3S-HS were intracellularly detected only in cells expressing either HS3ST2 or the tau mutation, or both, regardless of total tau levels (Fig. 5). Similarly, tau oligomers were only detected in cells accumulating intracellular 3S-HS. Interestingly, t-tau (K9JA) and tau oligomers (T22) signals colocalized with intracellular 3S-HS only in cells expressing HS3ST2, but not in cells expressing tau only (Fig. 5a–b), regardless of similar tau protein levels (Fig. 5c). Although the cells that accumulate the higher 3S-HS levels are those accumulating higher levels of tau oligomers, the effect in tau oligomerisation is likely more related to the extent of intracellular 3S-HS rather than the level of the sugar itself, as suggested by the absence of tau oligomers in HEK+tau cells, which accumulate 3S-HS at the cell membrane.

In cells expressing tau_P301S_, 3S-HS were systematically increased and intracellularly detected regardless of HS3ST2 expression although HS3ST2 expression increased tau oligomers levels compared to tau_P301S_ only expressing cells (Fig. 5b–h). The intracellular accumulation of 3S-HS in the HEK+tau_P301S_ cells supports that tau_P301S_ expression triggers the endogenous accumulation of these sugars without requirement of HS3ST2 overexpression (Fig. 5f–e). Together, these results indicate that 3S-HS intracellular location can be induced by simultaneous expression of both HS3ST2 and tau, by tau_P301S_ expression in cells (which endogenously produce some 3S-HS), or by over production of 3S-HS.

## Discussion

In a previous report we showed that HS3ST2 is critical for the abnormal phosphorylation of tau in AD-related tauopathy^28^. Here, we show for the first time that HS3ST2 expression promotes tau oligomerisation in cells expressing wild type tau or tau carrying the P301S mutation (tau_P301S_). Tau oligomers detection was assessed in the cultured cells and cell lysates by staining with the T22 antibody, which recognises oligomeric but not monomeric tau nor tau in advanced aggregational stages^39^. This agrees a role of intracellular 3S-HS in tau oligomerisation, T22 positive signal was only detected in cells expressing both HS3ST2 and tau (HEK+tau+HS3ST2 and HEK+tau_P301S_+HS3ST2), or in cells carrying tau_P301S_ only (HEK+tau_P301S_). In agreement with the presence of tau oligomers reported to be extractable in high salt (500 mM) Sarkosyl^35,36^, we observed tau multimers in stabilized conformations resistant to SDS^36,47^. However, as previously reported, it cannot be excluded that these multimers may be composed of tau in complex with other protein(s)^47,48^. Interestingly, P-tau oligomers were not only detected in the tau_P301S_ expressing cells (HEK+tau_P301S_+HS3ST2) but also in cells expressing the non-mutated tau and HS3ST2 (HEK+tau+HS3ST2), thus mimicking the tau aggregation process in both mutational^11,49^ and non-mutational tauopathies, which include AD^50^. P-tau signals levels were stronger when HS3ST2 was expressed together with tau and when 3S-HS accumulated inside cells. However, the signal decreased in HEK+tau_P301S_+HS3ST2 compared to HEK+tau+HS3ST2, suggesting lower T22 immunoreactivity in the last due to higher tau aggregation stages^11,51^, as confirmed by analysis of T22 in cells that could be analysed at earlier times of culture (made possible only by using bicistronic vectors). This agrees with a tau_P301S_ increased propensity to aggregate compared to native tau^51^. These results agree previous observations showing that suppression of HS3ST2 in a zebrafish model of tauopathy expressing human tau_P301L_ leads to tauopathy arrest and animal functional recovery^28^ and comfort the hypothesis that HS3ST2 participates to the cell autonomous P-tau oligomerisation and aggregation process in which intracellular 3S-HS might play a central role^22^.

The implication of 3S-HS in tau oligomerisation and aggregation is supported by the extensive use of heparin in the *in vitro* induction of native tau fibrils^14,16^. As heparin is a commercially available prototype of the endogenous 3S-HS^19^, it is not surprising that cells expressing both 3S-HS and tau are able to produce high levels of aggregated tau. Indeed, although heparin is produced predominantly in mast cells by HS3ST1^52^, other 3S-HS can also be produced by other HS3STs expressed in different tissues^19^, as HS3ST2 in brain^21,53,54^. Interestingly, regardless of the reported colocalization of HS with tau in the brain of AD in the 90’s^25–27^, the involvement of neural HS sulfotransferases in the development of tauopathy was until recently disregarded. This is possibly because HS are typically described to be located at the cell surface under physiological conditions^23,24^, and thus their intracellular accumulation in AD^25–27,55^ breaks the dogma of their extracellular location. Nevertheless, by using cell models of tauopathy, we previously showed that 3S-HS were first located at the cell membrane before internalization, delineating a link with the biology at the cell membrane^28^. Here, we show in the engineered HEK293 cells expressing HS3ST2 and tau, that 3S-HS intracellularly accumulate in cells that produce tau oligomers, whereas 3S-HS are predominantly located at the cell membrane in control wild type cells, in which tau oligomers were not detected (HEK Ctr cells). Indeed, by using FRET experiments, we previously showed that 3S-HS can physically interact with tau in cell models of tauopathy^28^, although aggregation was not assessed in that study. Moreover, the hypothesis of an intracellular 3S-HS induction of aggregation in a cell autonomous manner (without supplementing on tau seeds) is supported by previous proton nuclear magnetic resonance (^1^H NMR) studies showing that heparin can induce tau β-sheet conformations, characteristic of fibrillar protein aggregates^16^.

Accumulation of tau aggregates in neurons is an important pathological signature in multiple neurodegenerative disorders including AD, FTDP17, and other tauopathies^1,9^. Although great efforts have been made to understand the tau aggregation processes, there is still an imperative need to identify new key molecular players involved in this process, as well as to develop robust cellular systems to test the pertinence of these players in the research of new therapeutic candidates. Here, we show that tau aggregation can occur in a cell autonomous manner, as it happened without addition of exogenous inducers of aggregation in our engineered cells, which represent a new cell model of tauopathy. Indeed, in most cell models of tauopathy tau aggregation is induced by addition of exogenous tau seeds or tau seeds from reborning cells^56,57^, by addition of exogenous aggregation inducing molecules^58,59^, or by expression of tau carrying pathogenic mutations^28,60,61^. We used HEK293 cells since they are easily transfected and because they exhibit some characteristics of neuronal lineage cells^62^, including expression of more than 60 neuron-specific genes^63^. Moreover, these cells give opportunity to investigate the reciprocal effect of HS3ST2 and tau expression, independently of each other, given that they lack or show very low expression levels of HS3ST2 and tau. Thus, HEK293 cells were permanently transfected to express HS3ST2 alone or in association with tau or tau_P301S_. Interestingly, HS3ST2 protein levels remained undetectable in cells transfected with tau only (tau or tau_P301S_), indicating that tau has no effect in HS3ST2 expression, at least in the HEK293 cells. Accordingly, HS-related genes have not been reported in the panel of genes which expression increases after increasing tau expression^64^. As HS3ST2 and tau could be stably and robustly expressed in the HEK293 cell line, we additionally propose this model, or cells transfected with other HS3STs, as a convenient tool to investigate the involvement of 3S-HS in tau protein abnormal phosphorylation and pathologic oligomerisation. Taken together, our results show that 3S-HS produced by HS3ST2 can generate cell autonomous tau aggregation by starting from tau oligomerisation and evolving through tau aggregation in a time dependent manner. However, the mechanism driving the 3S-HS intracellular accumulation remains to be clarified.

## Conclusion

Cell autonomous hyperphosphorylation and oligomerisation of tau were induced through simultaneous expression of the neural HS3ST2 and tau in cultured cells, in which 3S-HS colocalized with tau at the intracellular level. This mimics the intracellular accumulation of HS observed in neurons of the AD^25–27,55^. As HS3ST2 is an enzyme predominantly expressed in brain regions vulnerable to AD, as cortex and hippocampus^33^, our results suggest the involvement of 3S-HS in the mechanisms leading to tau hyperphosphorylation, oligomerisation, and aggregation in the AD brain. Moreover, the cell autonomous production of tau aggregates in the absence of any tau mutation supports that 3S-HS can drive tau aggregation in cells that express tau and in which HS3ST2 is predominantly expressed, as in neural cells. Indeed, these cells are here mimicked by inducing the expression of tau and HS3ST2 in HEK cells, resulting in an original glycan-derived model of cell autonomous tauopathy. This new cell model offers the possibility to bypass tau aggregation inducers used to promote fibrillization of full-length wild-type tau^65^. Moreover, as HS accumulate in brain of AD at early stages of the disease, we propose that inhibiting the interaction of HS with tau will represent a breakthrough for developing new efficient therapeutic drugs for AD and related tauopathies. We are currently working in this area.

## Supplementary Information

Supplementary Information 1.

Supplementary Information 2.

Supplementary Information 3.
