# Supplementary material for: HS3ST2 expression induces the cell autonomous aggregation of tau
Source: Sci Rep. 2022 Jun 27;12:10850. doi: 10.1038/s41598-022-13486-6 (PMC9237029; doi:10.1038/s41598-022-13486-6)
Supplement: Supplementary file 1 — Supplementary Information 1. [file 41598_2022_13486_MOESM1_ESM.pdf]

Supplementary Figures 1-6

HS3ST2 EXPRESSION INDUCES THE CELL AUTONOMOUS AGGREGATION OF TAU

Huynh MB†[1], Rebergue N†[1], Merrick H[1], Gomez-Henao W[1,2], Jospin E[1],

Biard DSF*[1,3], Papy-Garcia D*

Supplementary Figure S1

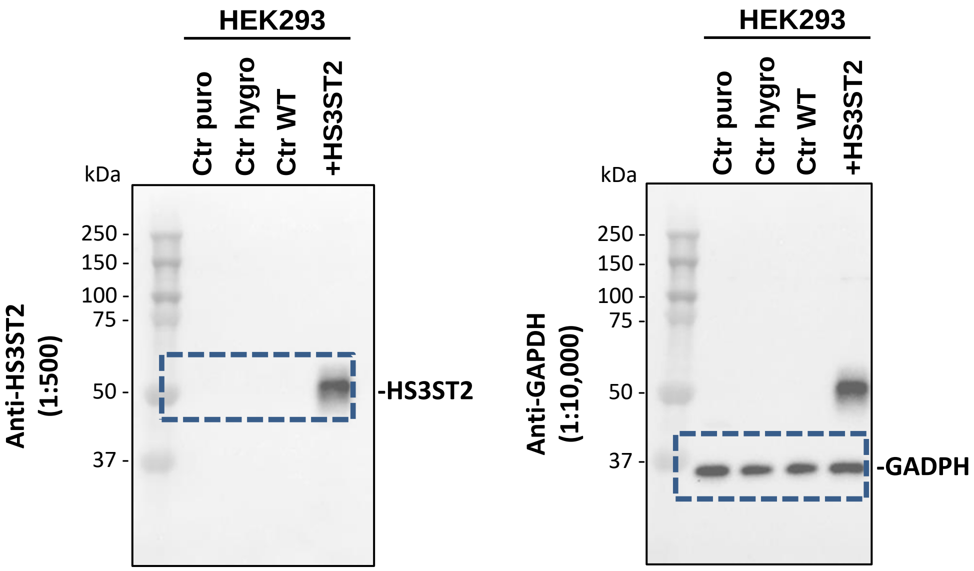

Supplementary Figure S1. Original full-length immunoblots of cell lysates shown in Figure 1c. HEK293 cells were transfected to express HS3ST2. SDS PAGE (4-8%) of soluble proteins (RIPA extracted) immunoassayed with anti-HS3ST2 (1:500) followed by GAPDH (anti-GAPDH, 1:10,000) as loading control. Cell lysates containing 1 µg of proteins (quantified with BCA assay) were loaded. All immunoblots were exposed for 120 sec. Dotted blue squares represent regions selected for main Figure 1c.

Supplementary
Figure S2

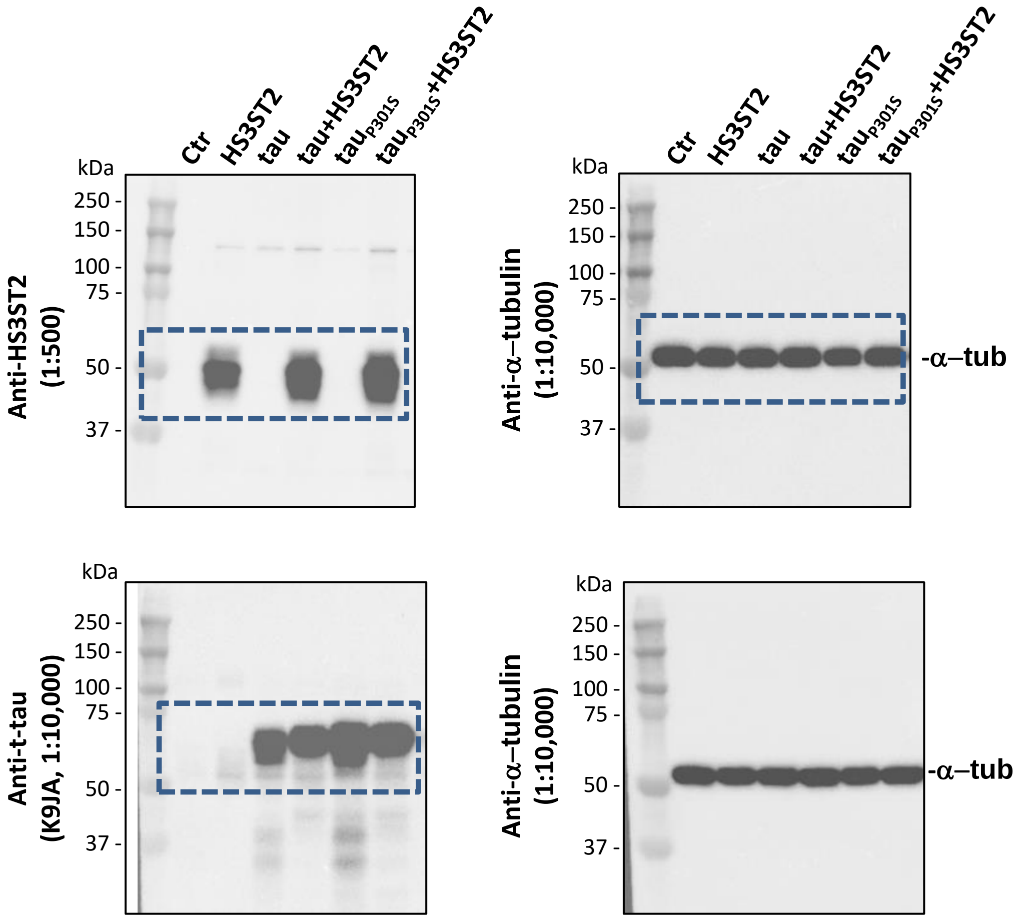

Supplementary Figure S2. Original full-length immunoblots of cell lysates shown in Figure 2e. HEK293 cells were transfected to express HS3ST2, tau, and HS3ST2 together with tau or tau$_{P301S}$. SDS PAGE (4-8%) of soluble proteins (RIPA extracted) immunoassayed with anti-HS3ST2 (1:500) or total tau (K9JA, 1:10,000), as indicated. α-tubulin (anti-α-tubulin, 1:10,000) was used as loading control in tau-stained blots. In HS3ST2 immunoblotted gels *α*-tubulin was loaded in different gels since it migrates at similar MW than HS3ST2 (approximately 50 kDa). Cell lysates containing 5 μg of proteins (quantified with BCA assay) were loaded. Immunoblots were exposed for 120 sec. Dotted blue squares represent regions selected for main Figure 2e.

Supplementary
Figure S3

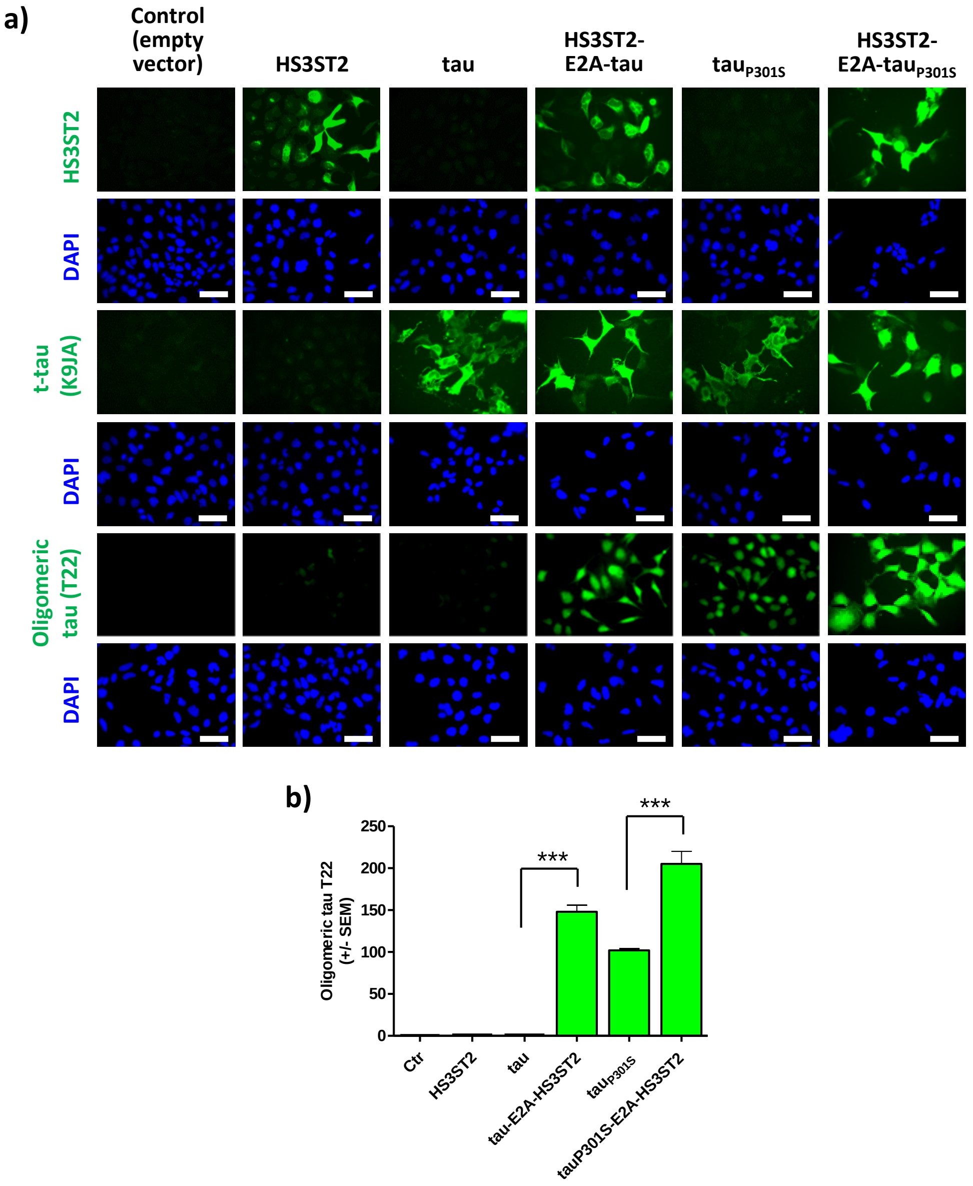

Supplementary Figure S3. HS3ST2, total tau and oligomeric tau ICC staining in HEK293 cells expressing HS3ST2 or/and tau or tauP301S 4 days after transfection. Cells were transfected with monocistronic or bicistronic (containing an E2A sequence) vectors to independently or simultaneously express HS3ST2 with tau or tauP301S. **a)** Immunostaining of HS3ST2, total tau (t-tau; K9JA antibody) or oligomeric tau (T22 antibody) in green. Cells were counterstained with DAPI to visualize nuclei (blue). Scale bar = 25 µm. b) More than 100 individual cells in at least 3 fields per sample were analysed with ImageJ and GraphPad Prism v5 (GraphPad Software, La Jolla, CA, USA) (mean + /- SEM).

Supplementary Figure S4

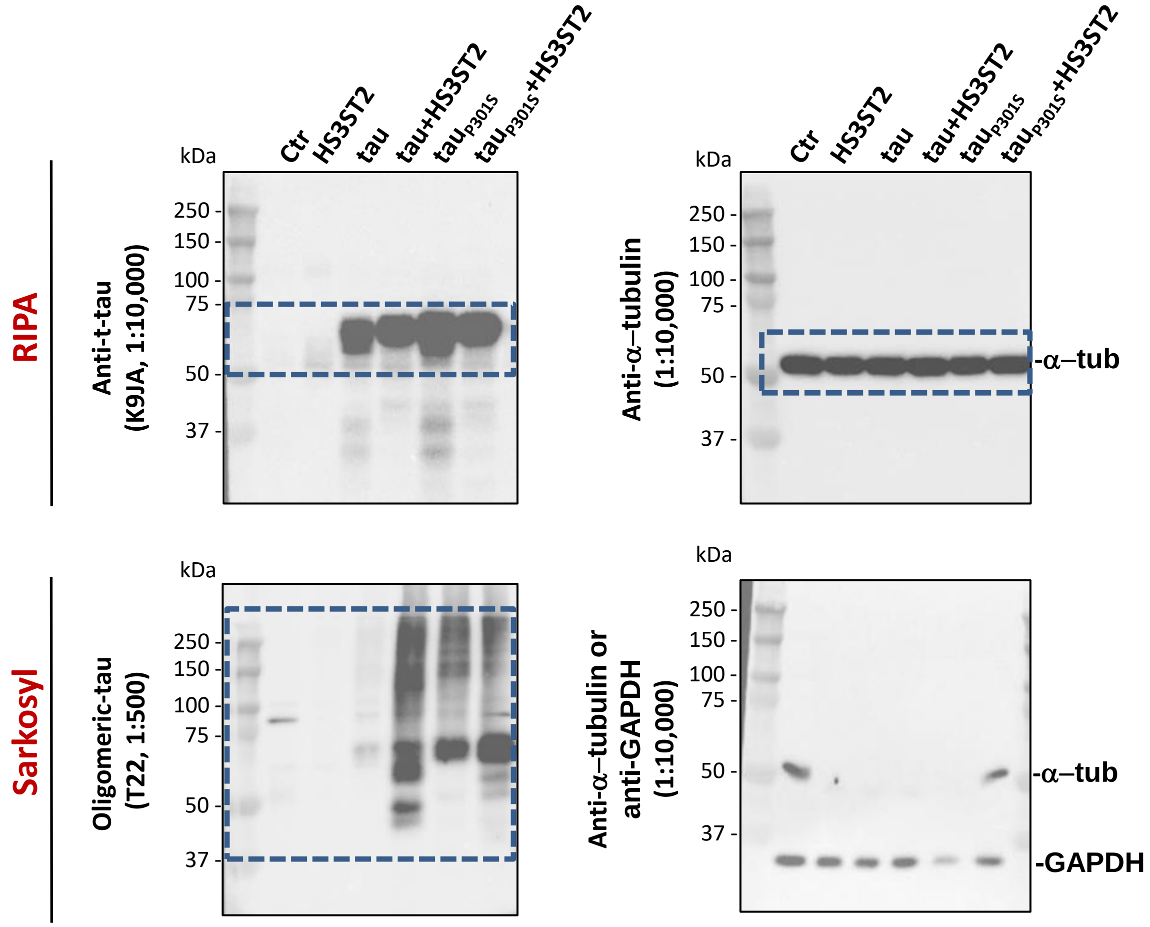

Supplementary Figure S4. Original full-length immunoblots of cell lysates shown in Figure 3c. HEK293 cells were transfected to express HS3ST2, tau, or HS3ST2 together with tau or tau$_{P301S}$. SDS PAGE (4-8%) analysis was performed with total tau (K9JA, 1:10,000) or oligomeric tau (T22, 1:500), as indicated. α-tubulin (anti-α-tubulin, 1:10,000) or GAPDH (anti-GAPDH, 1:10,000) were used as loading controls. Globally, α-tubulin was found to be appropriate as loading control for total tau (K9JA) under denaturating conditions. However, in high salt Sarkosyl oligomeric tau fractions, α-tubulin was hardly recovered possibly because low solubility in the high salt buffer. Similarly, in Sarkosyl non-denatured samples both α-tubulin and GAPDH were not stable, as here shown. Thus, loading control protein levels were controlled in denaturated samples. Cell lysates containing 5 µg of proteins (quantified with BCA assay) were loaded. Immunoblots were exposed for 120 sec. Dotted blue squares represent regions selected for main Figure 3c.

Supplementary Figure S5

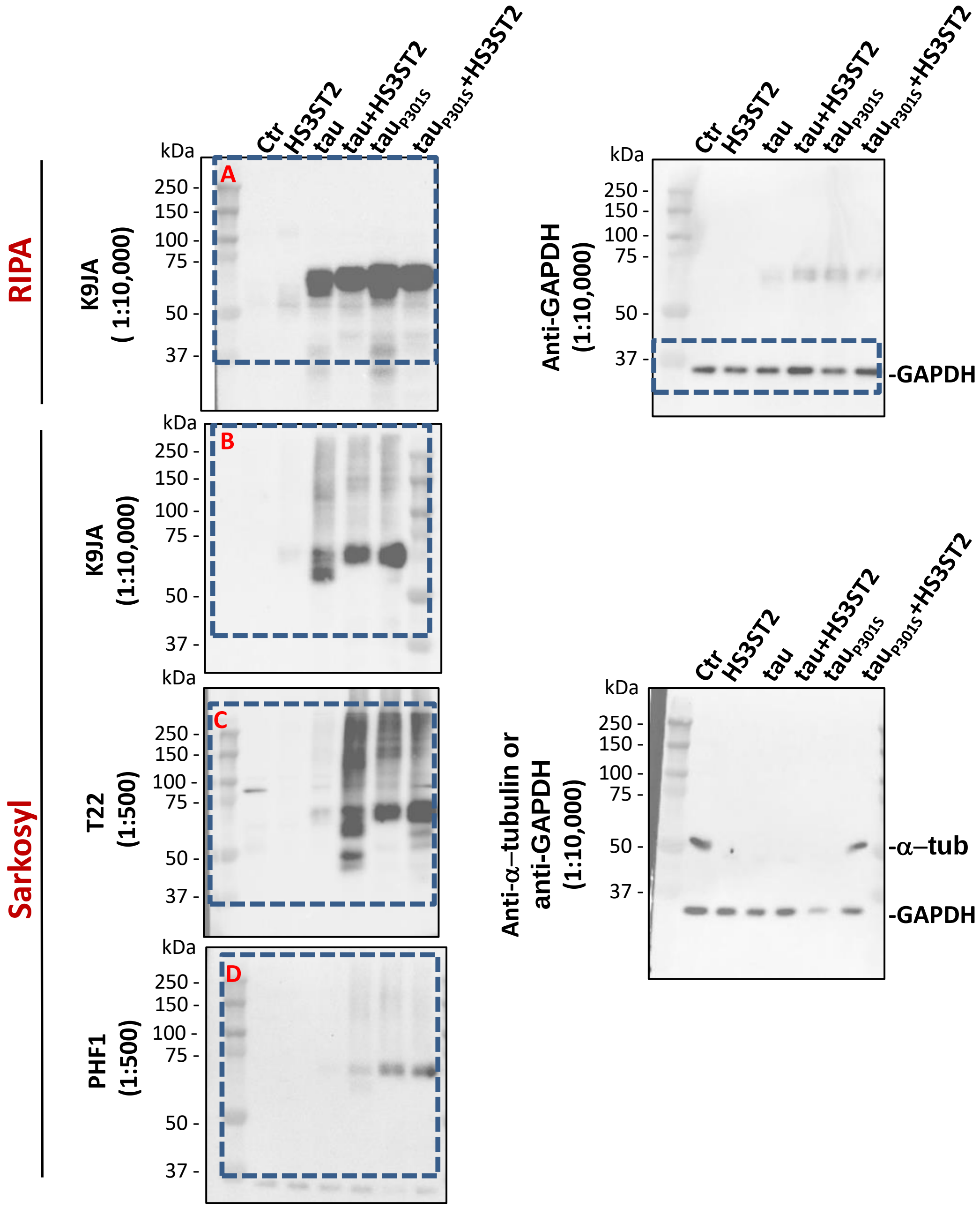

Supplementary Figure S5. Full-length immunoblots of cell lysates shown in Figure 4a. HEK293 cells were transfected to express HS3ST2, tau, or HS3ST2 together with tau or tau$_{P301S}$. SDS PAGE (4-8%) analysis was performed with: A, B: total tau (K9JA, 1:10,000), C: oligomeric tau (T22, 1:500), and D: P-tau (PHF1, 1:500), as indicated. α-tubulin (anti-α-tubulin, 1:10,000) or GAPDH (anti-GAPDH, 1:10,000) were used as loading control in separated blots to avoid tau oligomers interferences. It is to note that when samples in high salt Sarkosyl were immunoassayed with T22, any signal was observed for GAPDH nor for α-tubulin in the same blot, indicating difficulty to reveal control loading proteins in high salt Sarkosyl samples. Moreover, in non-denaturated samples immunoassayed with the anti P-tau PHF1 antibody a non-specific band appeared at the GAPDH MW. Thus, different blots had to be loaded for tau oligomers and for control loading proteins. Proteins in RIPA (A) were loaded with Laemmli. Proteins (5 µg, quantified with BCA assay) in Sarkosyl (B, C, and D) were loaded without Laemmli. Immunoblots were exposed for 120 sec. Dotted blue squares represent regions selected for main Figure 4a.

Supplementary Figure S6

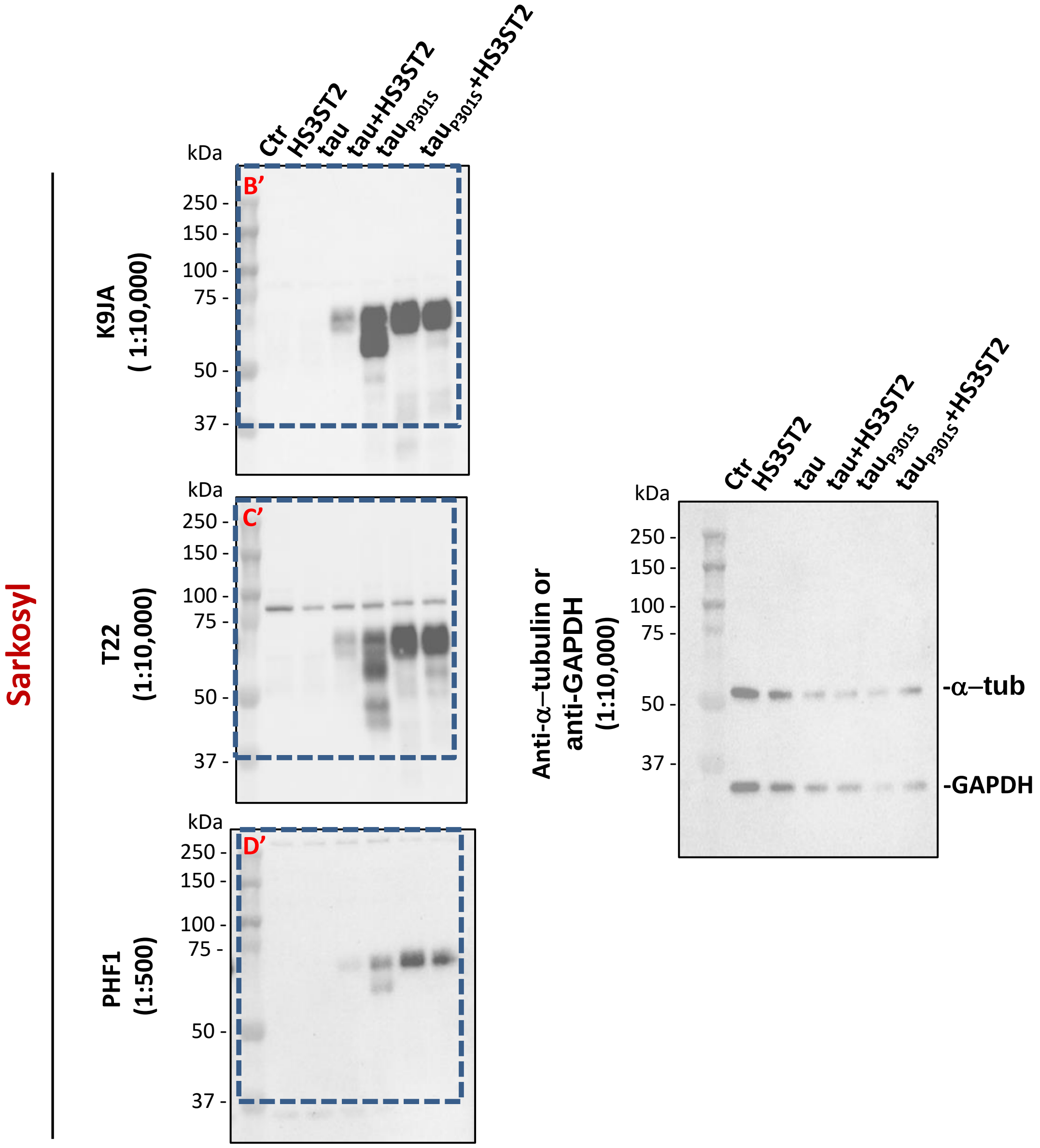

Supplementary Figure S6. Original full-length immunoblots of cell lysates shown in Figure 4a. HEK293 cells were transfected to express HS3ST2, tau, or HS3ST2 together with tau or tau_{P301S}. SDS PAGE (4-8%) analysis was performed as in Fig. S5, but here proteins in high salt Sarkosyl were loaded with Laemmli: B': total tau (K9JA, 1:10,000), C': oligomeric tau (T22, 1:500), and D': P-tau (PHF1, 1:500), as indicated. α-tubulin (anti-α-tubulin, 1:10,000) or GAPDH (anti-GAPDH, 1:10,000) were used as loading control under non denaturating conditions. 5 µg of proteins (quantified with BCA assay) were loaded. Immunoblots were exposed for 120 sec. Dotted blue squares represent regions selected for main Figure 4a.
