# Supplementary material for: HS3ST2 expression induces the cell autonomous aggregation of tau
Source: Sci Rep. 2022 Jun 27;12:10850. doi: 10.1038/s41598-022-13486-6 (PMC9237029; doi:10.1038/s41598-022-13486-6)
Supplement: Supplementary file 2 — Supplementary Information 2. [file 41598_2022_13486_MOESM2_ESM.pdf]

Supplementary Method 1

HS3ST2 EXPRESSION INDUCES THE CELL AUTONOMOUS AGGREGATION OF TAU

Huynh MB[†1], Rebergue N[†1], Merrick H[1], Gomez-Henao W[1,2], Jospin E[1], Biard DSF*[1,3], Papy-Garcia D*[1]

Bicistronic pEBV vector allowing simultaneous expression of HS3ST2 and tau

Plasmids carrying cDNA sequences allowing expression of human wild type tau (further referred as 'tau') and human tau carrying the mutation P301S (further referred as 'tau_{P301S}') in bacteria were kindly provided by Professor M. Goedert (University of Cambridge)[1]. Human full length HS3ST2 (NM_006043) was synthetized (Eurofins, France) and cloned in pcDNA™3.1 vectors (Invitrogen) for protein expression in bacteria. For expression independent or simultaneous expressions of tau and HS3ST2 in mammal cells, the corresponding cDNA sequences were included in replicative pEBV plasmids (Fig. SM-1)[2]. pEBV plasmids containing single transcription cartridges (HS3ST2, MAPT, or $MAPT_{P301S}$) and bicistronic transcription cartridges (HS3ST2 and MAPT or $MAPT_{P301S}$) were generated for expression under puromycin selection. In the bicistronic cartridges, the HS3ST2 and tau (or tau_{P301S}) coding sequences were separated by cDNA sequence coding for the equine rhinitis A virus self-cleaving E2A peptide, which allows simultaneous multi-gene expression without generation of fussed proteins[3]. Human cytomegalovirus (CMV) promoter sequence was included to drive transcription[2]. Construct sequences were confirmed by DNA sequencing

(Eurofins Genomics, results non-shown). Naming and main features of the generated plasmids are detailed in Table SM-1.

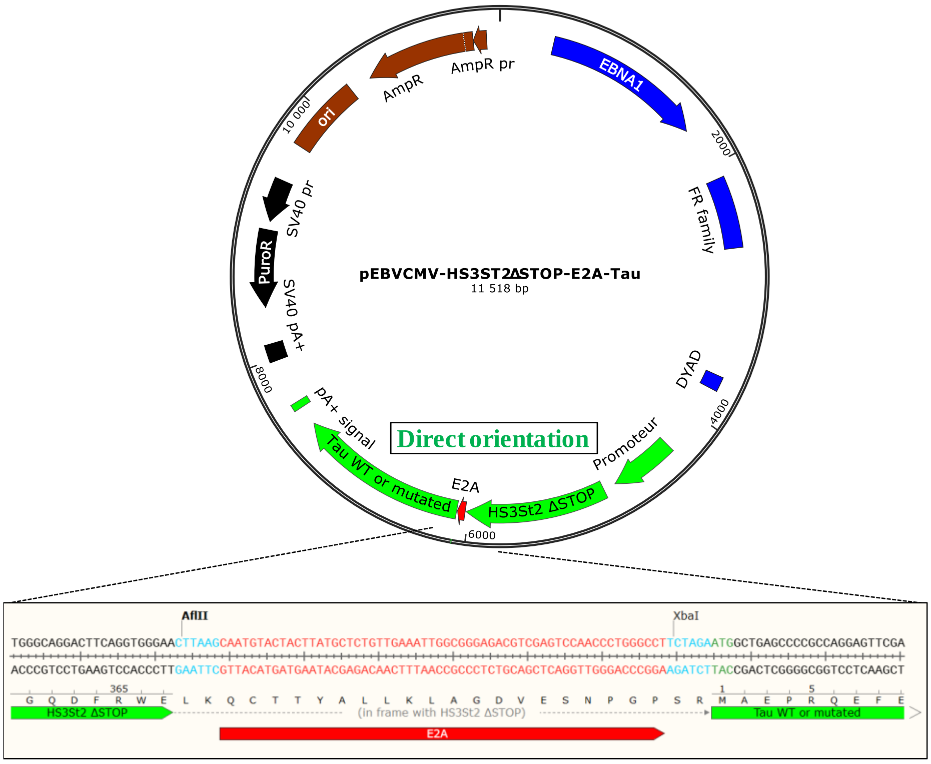

Figure SM-1. Physical map of a bicistronic pEBV vector allowing simultaneous expression of HS3ST2 and tau. Main features of replicative pEBV plasmids have been extensively described[2]. These vectors carry the CMV promoter for driving the transcription of HS3ST2 and/or tau. In bicistronic vectors, the HS3ST2 coding sequence (CDS), without its STOP codon, was in phase with a modified self-cleaving E2A peptide and the tau CDS (tau or tau_{P301S}). Sequences were annotated with the SnapGene software.

Stable transfection of HEK293 cells

Human embryonic kidney (HEK293) cells (provided by Dr A. Delaunay, CEA Saclay, France) were cultured in Dulbecco's Modified Eagle Medium (DMEM, Sigma) supplemented with 10% foetal calf serum (FCS), 100 U/mL penicillin, 100 µg/mL streptomycin, 2 mM glutamin, 10 mM HEPES (Sigma products), under 5% CO_2. For transfection experiments, approximately 100,000 cells were transfected with the agent JetPrime (Ozyme) following manufacturer's instructions. An empty pEBV vector was used to follow effects of transfection. For Sarkosyl experiments, 10^6 cells were transfected. From 24 hours (h) after starting transfection, cells were continually maintained under selection by supplementing the cell culture medium with 0.4 µg/mL puromycin (VWR). Cells were maintained in this medium

during different periods of time: short-term periods corresponded to 4-5 days in culture (1 passage), mid-term culture period corresponded to 14-16 days in culture (3-4 passages), and long-term culture periods corresponded to 40-44 days in culture (7-8 passages).

Table SM-1. Vectors, cloning primers, and cell names used in the study.

			Cloning primers		
**N° vector**	**Transgene**	**Puromycin pEBV plasmid**	**Forward primer**	**Reverse primer**	**Name of cells**
pBD3789	none	pEBVCMV	--	--	HEK-CTL
pBD3690	HS3ST2 (NM_006043)	pEBVCMV-HS3ST2	primer BD16_860F: ataccggtctagaATGGCCTATAGGGTCCTGGGCCGCGCG	primer BD13_663R: atgtcgacgcggccgcTTATTCCCACCTGAAGTCCTGCCCAAC	HEK-HS3ST2
pBD3793	tau (NM_005910)	pEBVCMV-tau	primer BD14_775F: ataccggtctagaATGGCTGAGCCCCGCCAGGAGTTCGAA	primer BD14_776R: atgtcgacgcggccgcTCACAAACCCTGCTTGGCCAGGGAGGC	HEK-tau
pBD3794	tauP301S (NM_005910)	pEBVCMV-tauP301S	primer BD14_775F: ataccggtctagaATGGCTGAGCCCCGCCAGGAGTTCGAA	primer BD14_776R: atgtcgacgcggccgcTCACAAACCCTGCTTGGCCAGGGAGGC	HEK-tau$_{P301S}$
pBD3770	HS3ST2 + Tau	pEBVCMV-HS3ST2/E2A/tau			HEK-HS3ST2/tau
pBD3810	HS3ST2 + TauP301S	pEBVCMV-HS3ST2/E2A/tauP301S			HEK-HS3ST2/tau$_{P301S}$

References

1. Falcon, B. *et al.* Conformation determines the seeding potencies of native and recombinant Tau aggregates. *J Biol Chem* 290, 1049–1065 (**2015**).

2. Biard, D. S., Cordier, A. & Sarasin, A. Establishment of a human cell line for the detection of demethylating agents. *Exp. Cell Res.* 200, 263–271 (**1992**).

3. Wang, Y., Wang, F., Wang, R., Zhao, P. & Xia, Q. 2A self-cleaving peptide-based multi-gene expression system in the silkworm Bombyx mori. *Sci Rep* 5, 16273 (**2015**).
