# Supplementary material for: HS3ST2 expression induces the cell autonomous aggregation of tau
Source: Sci Rep. 2022 Jun 27;12:10850. doi: 10.1038/s41598-022-13486-6 (PMC9237029; doi:10.1038/s41598-022-13486-6)
Supplement: Supplementary file 3 — Supplementary Information 3. [file 41598_2022_13486_MOESM3_ESM.pdf]

Supplementary Table 1

HS3ST2 EXPRESSION INDUCES THE CELL AUTONOMOUS AGGREGATION OF TAU

Huynh MB†[1], Rebergue N†[1], Merrick H[1], Gomez-Henao W[1,2], Jospin E[1],

Biard DSF*[1,3], Papy-Garcia D*[1]

Table S1. Primers used for HS3STs transcripts quantification by qPCR.

Gene symbol	Forward primer[a]	Reverse primer[a]	Amplification efficiency[b]	Amplicon size
HS3ST1	GAACGAGGTCCACTTCTTCG	TTCTCCACTGTGAGCTGGTG	1.92	104
HS3ST2	GGAACCCCACTTCTTTGACA	GTCGAGGAGCCTCTTGAGTG	2.10	133
HS3ST3A	TCACAGGTGGGGTCCAAG	GTTCTCTCCGCCCGAGTT	2.05	152
HS3ST3B	CATCATCGGACGGTCATCTT	AAACACGTGCCAGGACAAAT	1.93	157
HS3ST4	AAGAGCAAAGGTCGGACTCA	ACCCTCTTCCTGTTCCCACT	2.06	131
HS3ST5	CCTGCTTGAAATGCTGAACC	TACCACTCAATGCCCTTACC	1.95	101

[a] The qPCR forward and reverse primers were obtained from Eurofins.
[b] Amplification efficiency is determined using the formula $10^{-1/slope}$. For the actual calculations, the base of the exponential amplification function is used (e.g. 1.92 means 92% amplification efficiency).
